# 5,5′-Seleno­bis­(2-hy­droxy­benzaldehyde)

**DOI:** 10.1107/S1600536811042097

**Published:** 2011-10-22

**Authors:** Ming-Hu Wu, Wen-Ju Liu

**Affiliations:** a School of Chemistry and Chemical Engneering, Guangdong Pharmaceutical University, Guangzhou 510006, People’s Republic of China; b College of Chemistry, Central China Normal University, Wuhan 430079, People’s Republic of China

## Abstract

In the title mol­ecule, C_14_H_10_O_4_Se, the dihedral angle between the two benzene rings is 74.6 (1)°. Both hy­droxy­benzaldehyde groups form intra­molecular O—H⋯O hydrogen bonds. In the crystal, pairs of mol­ecules are linked by pairs of weak C—H⋯π(arene) inter­actions, forming centrosymmetric dimers. In addition, mol­ecules are linked by π–π stacking inter­actions, with a centroid–centroid distance of 3.785 (2) Å, forming chains along the *c* axis.

## Related literature

For background to organo-selenium compounds, see: Mukherjee *et al.* (2006 ▶); Phadnis *et al.* (2005 ▶); Braga *et al.* (2005 ▶); Mugesh *et al.* (2001 ▶).
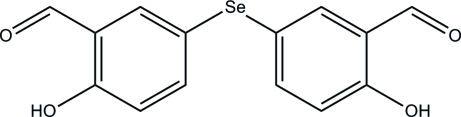


## Experimental

### 

#### Crystal data

C_14_H_10_O_4_Se
*M* *_r_* = 321.18
Monoclinic, 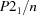

*a* = 7.7652 (5) Å
*b* = 11.9129 (8) Å
*c* = 13.3353 (9) Å
β = 90.304 (1)°
*V* = 1233.58 (14) Å^3^
*Z* = 4
Mo *K*α radiation
μ = 3.05 mm^−1^
*T* = 296 K
0.30 × 0.20 × 0.20 mm

#### Data collection

Bruker SMART APEX CCD diffractometer
Absorption correction: multi-scan (*SADABS*; Bruker, 2001 ▶) *T*_min_ = 0.461, *T*_max_ = 0.581
7045 measured reflections
2550 independent reflections
2041 reflections with *I* > 2σ(*I*)
*R*_int_ = 0.100

#### Refinement

*R*[*F* ^2^ > 2σ(*F* ^2^)] = 0.041
*wR*(*F* ^2^) = 0.122
*S* = 1.08
2550 reflections
172 parameters
H-atom parameters constrained
Δρ_max_ = 0.64 e Å^−3^
Δρ_min_ = −0.54 e Å^−3^

### 

Data collection: *SMART* (Bruker, 2001 ▶); cell refinement: *SAINT* (Bruker, 2001 ▶); data reduction: *SAINT*; program(s) used to solve structure: *SHELXS97* (Sheldrick, 2008 ▶); program(s) used to refine structure: *SHELXL97* (Sheldrick, 2008 ▶); molecular graphics: *PLATON* (Spek, 2009 ▶); software used to prepare material for publication: *PLATON*.

## Supplementary Material

Crystal structure: contains datablock(s) I, global. DOI: 10.1107/S1600536811042097/lh5352sup1.cif

Structure factors: contains datablock(s) I. DOI: 10.1107/S1600536811042097/lh5352Isup2.hkl

Supplementary material file. DOI: 10.1107/S1600536811042097/lh5352Isup3.cdx

Supplementary material file. DOI: 10.1107/S1600536811042097/lh5352Isup4.cml

Additional supplementary materials:  crystallographic information; 3D view; checkCIF report

## Figures and Tables

**Table 1:** Hydrogen-bond geometry (Å, °) *Cg* is the centroid of the C8-C13 ring.

*D*—H⋯*A*	*D*—H	H⋯*A*	*D*⋯*A*	*D*—H⋯*A*
O1—H1⋯O2	0.82	1.90	2.621 (4)	146
O3—H3*A*⋯O4	0.82	1.95	2.660 (4)	145
C10—H10⋯*Cg*^i^	0.93	2.89	3.763 (3)	158

Symmetry code: (i) 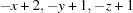.

**Fig. 1.**
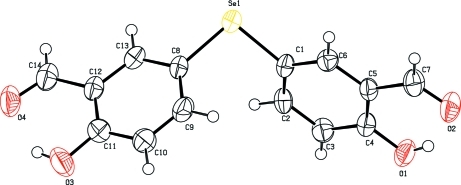
The molecular structure of (I) with 50% probability displacement ellipsoids for non-H atoms.

**Fig. 2.**
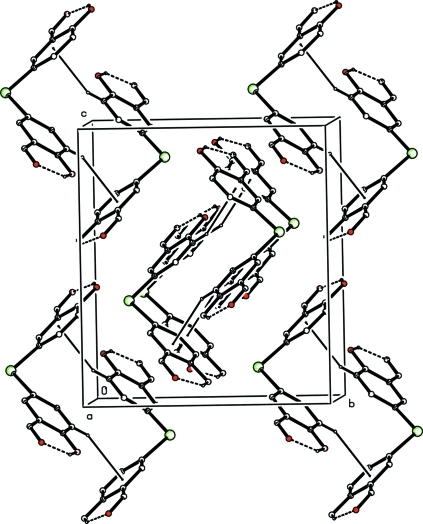
The crystal packing showing the hydrogen bonding interactions as thin solid lines. H atoms not involved in hydrogen bonds have been omitted.

**Fig. 3.**
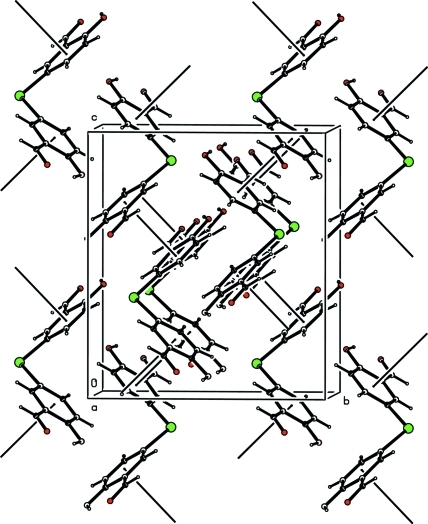
Part of the crystal structure showing π–π stacking interactions between benzene rings as thin solid lines.

## supplementary crystallographic information

### Comment

The organo-selenium nucleus is one of the most abundant structural nucleus found in natural products and biologically active molecules (*e.g.*, seleno-carbohydrates,selenoamino acids,and seleno-peptides)(Mukherjee *et al.*,2006; Phadnis *et al.*, 2005; Braga *et al.*, 2005). Moreover, organoselenium compounds have emerged as an exceptional class of structures that exemplify a role in biochemical processes, serving as important therapeutic compounds ranging from antiviral and anticancer agents to a variety of situations where free radicals are involved (Mugesh *et al.*, 2001). We are currently studing the synthesis of a new series of organoselenium compounds, such as selanes, diselenides and macrocyclic Schiff bases containing selenium atoms. Reported herein are the synthesis and X-ray structure of the title compound.

In the molecule (Fig. 1), the dihedral angle between the two benzene rings is 74.6 (1)°. Two intramolecular O—H···O hydrogen bonds are present in the molecule. The Se1—C1 and Se1—C8 bond lengths are the same within experimental error. The Se1—C1—C6—C5 and Se1—C8—C13—C12 torsional angles of -174.5 (2)° and -174.6 (2)°, respectively, indicate a slight deviation of the selenium atoms from the mean planes of the benzene rings.

In the crystal, pairs of molecules are linked by weak C—H···π (arene) interactions (see Table 1, Fig. 2). In addition, molecules are linked by *Cg*1···*Cg*2^ii^ (symmetry code (ii): -1/2+*x*, 1/2-y, 1/2+*z*) and *Cg*2···*Cg*1 ^iii^  (symmetry code (iii) : 1/2+*x*, 1/2-y, -1/2+*z*) π–π stacking interactions with a centroid-centroid distance of 3.785 (2)Å to form one-dimensional chains along the c axis (Fig. 3). Cg1 and Cg2 are the centroids of the C1-C6 and C8-C13 rings.

### Experimental

A mixture of salicylaldehyde (87.93 g, 0.72 mol), selenium dioxide (26.63 g, 0.24 mol)and concentrated hydrochloric acid (132 ml) was stirred for 0.5 h at room temperature. Then, the mixture was further stirred for 50 h at 353 K. The resulting reddish brown solid was filtered, washed with water and ethanol. The obtained yellowish solid was recrystallized with ethyl acetate and etanol (v:v=5:1) to give yellowish crystals of the title compound in yield 20.8%, which are suitable for X-ray analysis.

### Refinement

All H atoms were placed in calculated positions (C—H = 0.93 Å, O—H = 0.82Å) and included in a riding-model approximation, with *U*_iso_ (H) = 1.2*U*_iso_ (C) or 1.5*U*_iso_ (O)

### Crystal data

**Table:**

C_14_H_10_O_4_Se	*F*(000) = 640
*M**_r_* = 321.18	*D*_x_ = 1.729 Mg m^−^^3^
Monoclinic, *P*2_1_/*n*	Mo *K*α radiation, λ = 0.71073 Å
Hall symbol: -P 2yn	Cell parameters from 3183 reflections
*a* = 7.7652 (5) Å	θ = 2.3–27.8°
*b* = 11.9129 (8) Å	µ = 3.05 mm^−^^1^
*c* = 13.3353 (9) Å	*T* = 296 K
β = 90.304 (1)°	Block, yellow
*V* = 1233.58 (14) Å^3^	0.30 × 0.20 × 0.20 mm
*Z* = 4	

### Data collection

**Table:**

Bruker SMART APEX CCD diffractometer	2550 independent reflections
Radiation source: fine-focus sealed tube	2041 reflections with *I* > 2σ(*I*)
graphite	*R*_int_ = 0.100
φ and ω scans	θ_max_ = 26.5°, θ_min_ = 2.3°
Absorption correction: multi-scan (*SADABS*; Bruker, 2001)	*h* = −9→9
*T*_min_ = 0.461, *T*_max_ = 0.581	*k* = −10→14
7045 measured reflections	*l* = −16→16

### Refinement

**Table:**

Refinement on *F*^2^	Primary atom site location: structure-invariant direct methods
Least-squares matrix: full	Secondary atom site location: difference Fourier map
*R*[*F*^2^ > 2σ(*F*^2^)] = 0.041	Hydrogen site location: inferred from neighbouring sites
*wR*(*F*^2^) = 0.122	H-atom parameters constrained
*S* = 1.08	*w* = 1/[σ^2^(*F*_o_^2^) + (0.0633*P*)^2^] where *P* = (*F*_o_^2^ + 2*F*_c_^2^)/3
2550 reflections	(Δ/σ)_max_ = 0.001
172 parameters	Δρ_max_ = 0.64 e Å^−^^3^
0 restraints	Δρ_min_ = −0.54 e Å^−^^3^

### Fractional atomic coordinates and isotropic or equivalent isotropic displacement parameters (Å^2^)

**Table:**

	*x*	*y*	*z*	*U*_iso_*/*U*_eq_	
Se1	0.10731 (4)	0.81234 (3)	0.62308 (2)	0.04535 (18)	
C8	0.0015 (4)	0.7027 (3)	0.7079 (2)	0.0347 (7)	
C1	0.2434 (4)	0.7156 (3)	0.5393 (2)	0.0334 (6)	
C5	0.5117 (4)	0.6774 (2)	0.4565 (2)	0.0340 (7)	
C6	0.4134 (4)	0.7406 (3)	0.5238 (2)	0.0356 (7)	
H6	0.4640	0.8000	0.5583	0.043*	
O1	0.5229 (3)	0.5247 (2)	0.33974 (17)	0.0514 (6)	
H1	0.6219	0.5483	0.3355	0.077*	
C13	−0.1686 (4)	0.7131 (3)	0.7323 (2)	0.0369 (7)	
H13	−0.2337	0.7699	0.7028	0.044*	
C3	0.2627 (4)	0.5615 (3)	0.4229 (2)	0.0412 (7)	
H3	0.2116	0.5011	0.3901	0.049*	
O2	0.7856 (3)	0.6539 (2)	0.38446 (19)	0.0577 (7)	
O4	−0.5065 (3)	0.5951 (2)	0.8838 (2)	0.0593 (7)	
C4	0.4354 (4)	0.5874 (3)	0.4060 (2)	0.0350 (7)	
C12	−0.2473 (4)	0.6400 (3)	0.8007 (2)	0.0366 (7)	
C2	0.1688 (4)	0.6257 (3)	0.4884 (2)	0.0389 (7)	
H2	0.0534	0.6089	0.4990	0.047*	
C7	0.6922 (4)	0.7058 (3)	0.4396 (3)	0.0443 (8)	
H7	0.7378	0.7674	0.4733	0.053*	
C10	0.0214 (4)	0.5417 (3)	0.8167 (3)	0.0504 (9)	
H10	0.0866	0.4834	0.8436	0.060*	
C9	0.0956 (4)	0.6158 (3)	0.7512 (3)	0.0463 (8)	
H9	0.2115	0.6077	0.7354	0.056*	
C11	−0.1501 (4)	0.5530 (3)	0.8433 (3)	0.0456 (8)	
O3	−0.2165 (3)	0.4793 (2)	0.9087 (2)	0.0676 (8)	
H3A	−0.3182	0.4942	0.9183	0.101*	
C14	−0.4287 (4)	0.6536 (3)	0.8254 (3)	0.0490 (8)	
H14	−0.4889	0.7113	0.7941	0.059*	

### Atomic displacement parameters (Å^2^)

**Table:**

	*U*^11^	*U*^22^	*U*^33^	*U*^12^	*U*^13^	*U*^23^
Se1	0.0460 (2)	0.0397 (2)	0.0505 (3)	0.00471 (13)	0.01921 (17)	−0.00162 (15)
C8	0.0277 (15)	0.0413 (18)	0.0351 (15)	0.0014 (12)	0.0057 (12)	−0.0033 (13)
C1	0.0240 (14)	0.0430 (16)	0.0332 (15)	0.0034 (12)	0.0040 (12)	−0.0008 (14)
C5	0.0275 (15)	0.0406 (17)	0.0339 (14)	−0.0006 (12)	0.0046 (12)	0.0039 (13)
C6	0.0374 (16)	0.0373 (17)	0.0320 (15)	−0.0022 (13)	0.0003 (13)	−0.0048 (14)
O1	0.0497 (13)	0.0560 (15)	0.0485 (13)	0.0027 (11)	0.0165 (11)	−0.0142 (12)
C13	0.0354 (16)	0.0412 (17)	0.0341 (15)	0.0065 (13)	0.0019 (13)	−0.0070 (14)
C3	0.0424 (17)	0.0436 (18)	0.0376 (16)	−0.0076 (14)	0.0034 (14)	−0.0079 (15)
O2	0.0352 (14)	0.0726 (17)	0.0653 (16)	0.0044 (12)	0.0168 (12)	−0.0026 (15)
O4	0.0416 (14)	0.0705 (18)	0.0661 (16)	−0.0140 (12)	0.0206 (12)	−0.0110 (14)
C4	0.0287 (14)	0.0457 (18)	0.0306 (14)	0.0029 (13)	0.0023 (12)	0.0015 (14)
C12	0.0291 (15)	0.0434 (18)	0.0373 (16)	−0.0036 (13)	0.0032 (13)	−0.0113 (14)
C2	0.0253 (15)	0.050 (2)	0.0410 (17)	−0.0050 (13)	0.0053 (13)	0.0005 (15)
C7	0.0268 (16)	0.058 (2)	0.0483 (18)	−0.0035 (14)	0.0002 (14)	0.0020 (17)
C10	0.0308 (16)	0.049 (2)	0.072 (2)	0.0094 (15)	0.0054 (16)	0.0137 (19)
C9	0.0317 (16)	0.050 (2)	0.058 (2)	0.0061 (14)	0.0081 (15)	−0.0037 (17)
C11	0.0453 (18)	0.0418 (19)	0.0496 (18)	−0.0030 (15)	0.0058 (15)	−0.0008 (17)
O3	0.0511 (15)	0.0664 (18)	0.086 (2)	−0.0042 (13)	0.0179 (14)	0.0289 (16)
C14	0.0383 (19)	0.058 (2)	0.0504 (19)	−0.0010 (16)	0.0074 (16)	−0.0098 (19)

### Geometric parameters (Å, °)

**Table:**

Se1—C8	1.916 (3)	C3—C4	1.395 (4)
Se1—C1	1.925 (3)	C3—H3	0.9300
C8—C13	1.368 (4)	O2—C7	1.206 (4)
C8—C9	1.391 (4)	O4—C14	1.209 (4)
C1—C6	1.370 (4)	C12—C11	1.401 (5)
C1—C2	1.392 (4)	C12—C14	1.457 (4)
C5—C4	1.396 (4)	C2—H2	0.9300
C5—C6	1.401 (4)	C7—H7	0.9300
C5—C7	1.461 (4)	C10—C9	1.370 (5)
C6—H6	0.9300	C10—C11	1.386 (5)
O1—C4	1.344 (4)	C10—H10	0.9300
O1—H1	0.8200	C9—H9	0.9300
C13—C12	1.403 (5)	C11—O3	1.343 (4)
C13—H13	0.9300	O3—H3A	0.8200
C3—C2	1.374 (4)	C14—H14	0.9300
			
C8—Se1—C1	99.96 (14)	C11—C12—C13	119.1 (3)
C13—C8—C9	118.3 (3)	C11—C12—C14	120.6 (3)
C13—C8—Se1	119.7 (2)	C13—C12—C14	120.2 (3)
C9—C8—Se1	121.8 (2)	C3—C2—C1	121.2 (3)
C6—C1—C2	119.5 (3)	C3—C2—H2	119.4
C6—C1—Se1	119.3 (2)	C1—C2—H2	119.4
C2—C1—Se1	121.0 (2)	O2—C7—C5	123.8 (3)
C4—C5—C6	119.4 (3)	O2—C7—H7	118.1
C4—C5—C7	120.6 (3)	C5—C7—H7	118.1
C6—C5—C7	120.0 (3)	C9—C10—C11	120.5 (3)
C1—C6—C5	120.5 (3)	C9—C10—H10	119.7
C1—C6—H6	119.7	C11—C10—H10	119.7
C5—C6—H6	119.7	C10—C9—C8	121.5 (3)
C4—O1—H1	109.5	C10—C9—H9	119.3
C8—C13—C12	121.5 (3)	C8—C9—H9	119.3
C8—C13—H13	119.2	O3—C11—C10	118.4 (3)
C12—C13—H13	119.2	O3—C11—C12	122.6 (3)
C2—C3—C4	119.5 (3)	C10—C11—C12	119.0 (3)
C2—C3—H3	120.2	C11—O3—H3A	109.5
C4—C3—H3	120.2	O4—C14—C12	124.7 (4)
O1—C4—C3	118.2 (3)	O4—C14—H14	117.7
O1—C4—C5	121.9 (3)	C12—C14—H14	117.7
C3—C4—C5	119.9 (3)		
			
C1—Se1—C8—C13	−138.5 (3)	C8—C13—C12—C14	179.7 (3)
C1—Se1—C8—C9	45.8 (3)	C4—C3—C2—C1	−0.8 (5)
C8—Se1—C1—C6	−131.5 (3)	C6—C1—C2—C3	0.0 (5)
C8—Se1—C1—C2	53.3 (3)	Se1—C1—C2—C3	175.2 (2)
C2—C1—C6—C5	0.8 (5)	C4—C5—C7—O2	−1.6 (5)
Se1—C1—C6—C5	−174.5 (2)	C6—C5—C7—O2	178.4 (3)
C4—C5—C6—C1	−0.9 (5)	C11—C10—C9—C8	−1.3 (6)
C7—C5—C6—C1	179.2 (3)	C13—C8—C9—C10	0.1 (5)
C9—C8—C13—C12	1.3 (5)	Se1—C8—C9—C10	175.9 (3)
Se1—C8—C13—C12	−174.6 (2)	C9—C10—C11—O3	−179.1 (3)
C2—C3—C4—O1	−178.4 (3)	C9—C10—C11—C12	1.1 (6)
C2—C3—C4—C5	0.7 (5)	C13—C12—C11—O3	−179.5 (3)
C6—C5—C4—O1	179.2 (3)	C14—C12—C11—O3	−0.7 (5)
C7—C5—C4—O1	−0.8 (5)	C13—C12—C11—C10	0.2 (5)
C6—C5—C4—C3	0.1 (4)	C14—C12—C11—C10	179.0 (3)
C7—C5—C4—C3	−179.9 (3)	C11—C12—C14—O4	1.6 (5)
C8—C13—C12—C11	−1.4 (5)	C13—C12—C14—O4	−179.5 (3)

### Hydrogen-bond geometry (Å, °)

**Table:**

Cg is the centroid of the C8-C13 ring.

**Table:**

*D*—H···*A*	*D*—H	H···*A*	*D*···*A*	*D*—H···*A*
O1—H1···O2	0.82	1.90	2.621 (4)	146.
O3—H3A···O4	0.82	1.95	2.660 (4)	145.
C10—H10···Cg^i^	0.93	2.89	3.763 (3)	158

Symmetry codes: (i) −*x*+2, −*y*+1, −*z*+1.
